# Tibial Tubercle Avulsion Fracture with Multiple Concomitant Injuries in an Adolescent Male Athlete

**DOI:** 10.1155/2018/1070628

**Published:** 2018-08-02

**Authors:** Avinesh Agarwalla, Richard Puzzitiello, Austin V. Stone, Brian Forsythe

**Affiliations:** Midwest Orthopaedics at Rush, Rush University Medical Center, Chicago, IL, USA

## Abstract

Tibial tubercle avulsion fractures are an uncommon injury occurring due to strong contraction of the quadriceps femoris muscle during leg extension, ultimately causing failure of the physis at the patellar tendon insertion. This injury has been previously reported with various concomitant injuries, such as compartment syndrome from bleeding into the anterior compartment, vascular injury, patellar tendon avulsion, and meniscal injury—exhibited only with fracture types that extend intra-articularly. We report the case of a 14-year-old healthy adolescent male basketball player who sustained this injury as a result of a collision with another player. He initially reported to the emergency department and then presented to our practice, where he was diagnosed with a tibial tubercle avulsion fracture with patellar tendon rupture. During the operative management of these injuries, it was noted that fascial tissue avulsed through the injury site causing subacute extensive bleeding within the anterolateral compartments. Due to concerns of compartment syndrome, a fascial release was performed along the anterolateral compartments. By five months postoperatively, the patient demonstrated near-normal function, no evidence of extensor lag, and nearly full range of motion. Unlike previously reported cases, this is the first report of a patient who suffered such an injury with multiple concomitant injuries to the neighboring structures. Due to the severity of compartment syndrome and the variability in its temporal presentation from the initial injury, it is paramount that careful evaluation of vascular integrity and a low threshold for fasciotomy be in place to prevent vascular compromise.

## 1. Introduction

Tibial tubercle avulsion fractures occur in 0.4–2.7% of epiphyseal injuries and less than 1% of physeal injuries [1, 2]. This injury is most commonly seen in adolescent males during athletic activity but may also be associated in patients with osteogenesis imperfecta and Osgood-Schlatter disease [3, 4]. Strong contraction of the quadriceps femoris muscle during leg extension causes failure of the physis at the patellar tendon insertion [5].

Tibial tubercle avulsion fractures are managed operatively in most cases, and long-term outcomes are favorable with the resumption of sport to the previous level [4]. The high energy nature of the athletic injury is associated with compartment syndrome from bleeding into the anterior compartment, vascular injury [6], patellar tendon avulsion [7], and meniscal injury—exhibited only with fracture types that extend intra-articularly [8]. While there are various concomitant injuries, patients experiencing more than one concurrent injury with tibial tubercle avulsion fracture have not been reported in the literature.

In this report, we present a 14-year-old male who experienced sharp pain in his knee while jumping and colliding with another player during a basketball game. He was diagnosed with a displaced tibial tubercle avulsion fracture with proximal extension into the knee joint (Ogden type IIIB), as well as a patellar tendon avulsion. The injuries were managed with an open reduction internal fixation (ORIF) of the tibial tubercle and distal patellar tendon repair. Intraoperatively, it was noted that extensive bleeding accumulated subacutely within the anterior and lateral compartments. There was sufficient concern for impending compartment syndrome which led to fascial compartment release.

## 2. Case Report

**Figure 1:**
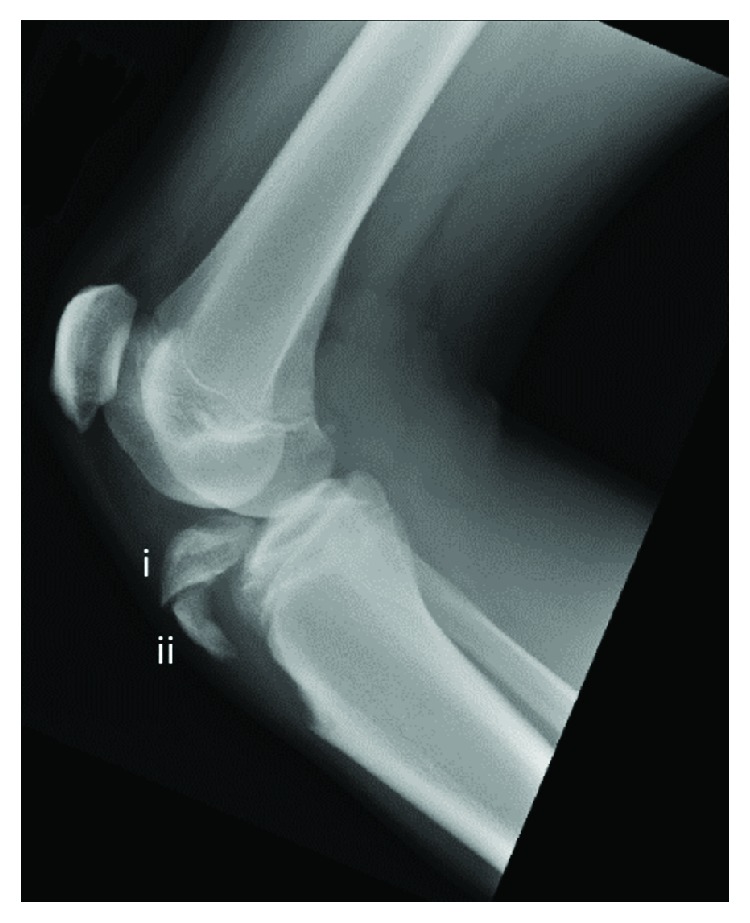
Preoperative lateral X-ray with knee at 30° flexion demonstrated tibial tubercle avulsion fracture extending into the joint space with two primary fragments: (i) anterior tibial plateau and (ii) tibial tubercle.

This patient is a 14-year-old male, who felt a popping sensation and significant right knee pain while jumping and colliding with another player during a basketball game the previous day. Following the injury, he was evaluated in an outside emergency department, where anterior, posterior, and lateral radiographs obtained in the emergency department demonstrated a tibial fracture consisting of two primary components (Figure 1). He was placed in a cast and sought a second opinion regarding findings and management.

**Figure 2:**
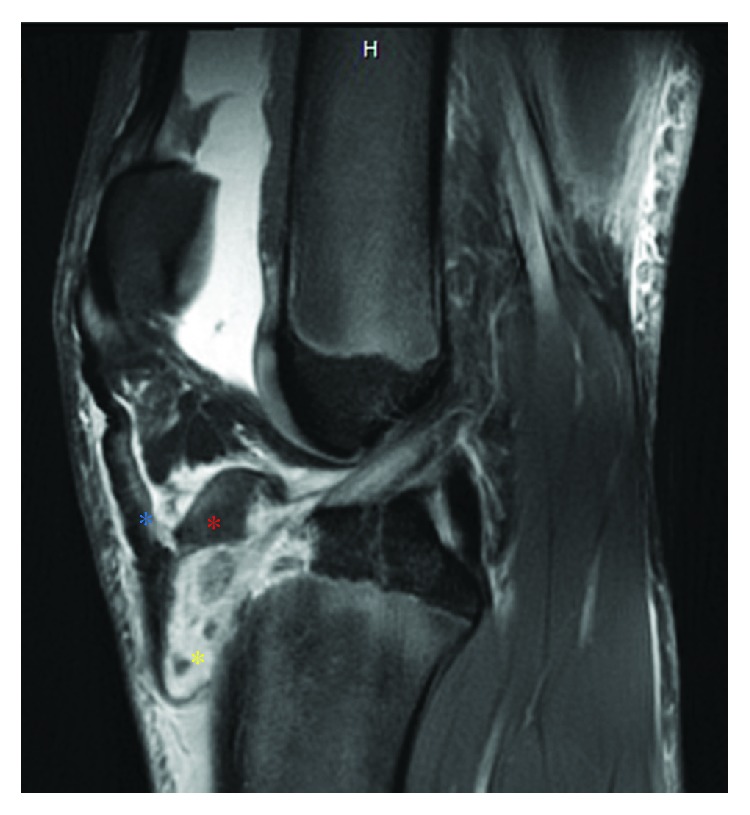
Preoperative sagittal view of MRI demonstrating tibial tubercle avulsion fracture (red asterisk), distal patellar tendon rupture (blue asterisk), and hematoma formation (yellow asterisk) at the site of injury.

Upon presentation to the clinic the following day, he reported mild pain (3/10) and noted no normal function of his leg. A physical exam was performed but was limited due to pain. Following the review of radiographic imaging, an MRI was performed, which demonstrated a type IIIB tibial tubercle avulsion fracture and complete tear of the patellar tendon from its distal attachment site, as well as a hematoma at the fracture site (Figure 2). After discussing the findings with the family, the patient was scheduled to undergo open reduction internal fixation of a type IIIB fracture and repair of the patellar tendon three days following the initial injury.

An 8-centimeter anterior incision was made at the superior aspect of the tibial tubercle and extended distally. At the patellar tendon insertion site on the tibia, the tendon was noted to be completely avulsed from the bone cortex distally, while proximally, the tendon remained attached to the displaced tubercle. The tendon remained attached to the inferior pole of the patella. The anterior tibial plateau fragment was anatomically reduced using two fully threaded noncannulated screws (Arthrex, Naples, FL), while the tibial tubercle fragment was reduced via bicortical fixation with a 50 mm fully threaded 3.5 mm cortical screw (Arthrex, Naples, FL).

The distal patellar tendon was completely avulsed through two-thirds of its length. To restore the native footprint of the patellar tendon, a 4.5 mm PEEK (polyetheretherketone) corkscrew anchor (Arthrex, Naples, FL) was placed slightly lateral to the anatomic insertion site to avoid a stress riser on the anterior tibial cortex. The anatomic repair of the patellar tendon was completed with two mattress sutures and tied.

**Figure 3:**
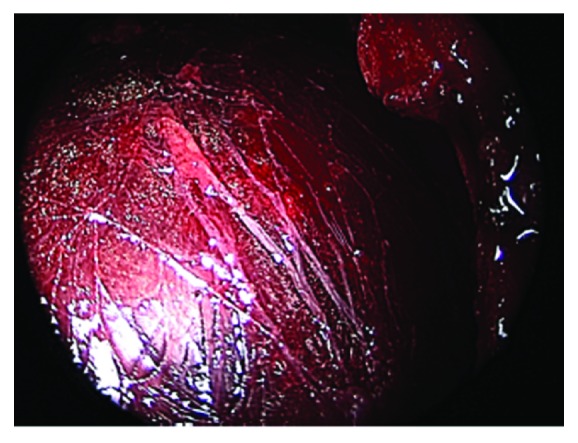
Intraoperative arthroscopic view of avulsed fascia and tibialis anterior muscle belly through the site of injury.

In addition to the avulsion of the patellar tendon and periosteum, it was noted that fascial tissue with tibialis anterior muscle belly avulsed through the injury site causing subacute extensive bleeding within the anterolateral compartments (Figure 3). This scenario raised concern for impending compartment syndrome, and an anterolateral compartment release was planned.

**Figure 4:**
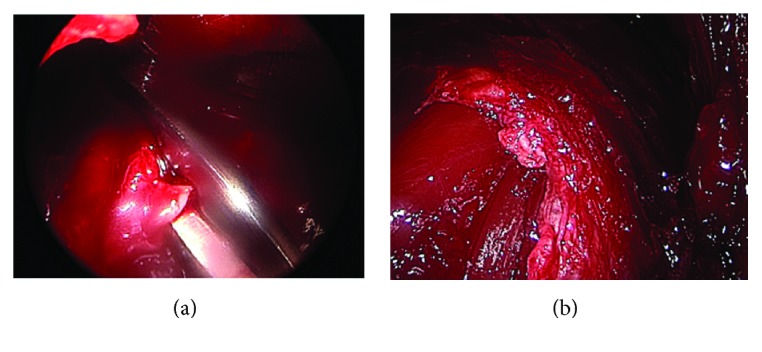
Intraoperative arthroscopic view of Metzenbaum scissors releasing fascial tissue (a) and the site of injury following fascial tissue release (b).

Three 3-centimeter incisions were made along the anterolateral aspect of the leg. The first was located 3 centimeters distal to the neck of the fibula, the second was located 10 centimeters above the distal fibula tip, and the third was located at the midpoint between the two. Under endoscopic visualization, the intramuscular septum was identified and Metzenbaum scissors were used to cut through the fascial compartment beginning in the anterior compartment and extending proximally then distally to the midtibia (Figure 4). The fascial incision was extended posteriorly into the peroneal compartment and then was extended proximally and distally to the midtibia. These steps were repeated for the midpoint and distal incision sites. Distally, the course of the superficial perineal nerve was identified and the nerve itself was protected during the distal release of the anterior compartment. It was believed that the impending compartment syndrome occurred due to damage to the surrounding bony and muscular tissue. A medium Hemovac drain was placed along the length of the lateral compartment, exiting in the posterolateral proximal leg. The patient was placed in a hinged knee brace which was locked in extension. He was discharged home later that day.

**Figure 5:**
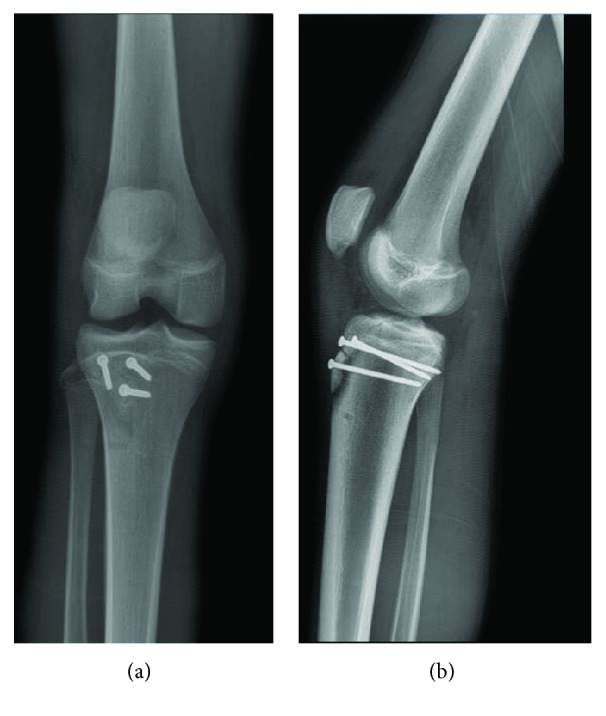
Anterior-posterior (a) and lateral (b) X-rays 2.5 weeks following operative management demonstrates well-positioned screws with callus formation beginning at the site of injury.

On postoperative day number two, the patient's Hemovac drain was removed by a family member. The patient was seen 1 week postoperatively and noted moderate pain (6/10) and 0% normal function. On physical examination, incisional sites were clean, dry, and intact and a small fracture blister was noted on the posterior aspect of the knee—which was cleaned and redressed. Radiographic imaging revealed well-positioned screws, no evidence of new fractures or foreign bodies, and early evidence of callus formation. Two and a half weeks after surgery, the patient presented to the clinic for evaluation. He reported that he had no pain (0/10) and had 5% of his normal function at this time. On physical examination, he noted no tenderness to palpation of the knee joint, and he had 40 degrees of knee flexion. Anterior-posterior and lateral X-rays were taken which showed evidence of callus formation in the bone (Figure 5). At this time, it was recommended that the patient begin gentle active range of motion exercises with extension and light flexion. He was also encouraged to become full weight-bearing with the brace until its removal two months postoperatively.

**Figure 6:**
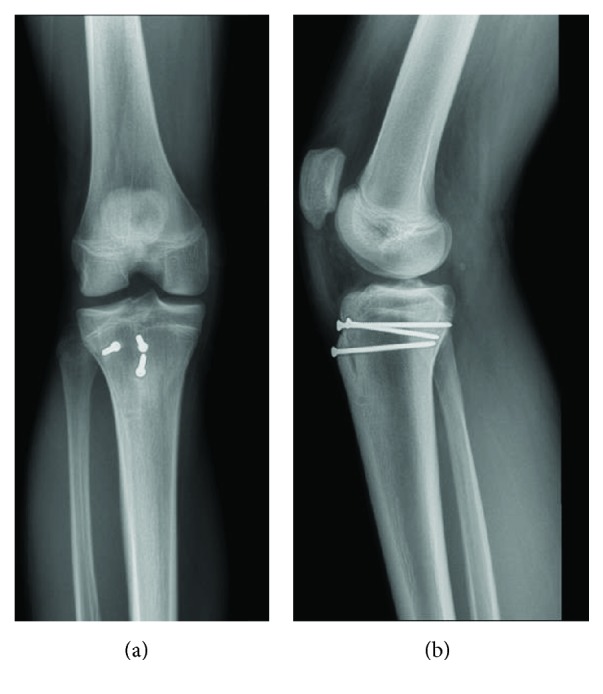
Anterior-Posterior (a) and lateral (b) X-rays 6 months following operative management demonstrates well-positioned screws with callus formation at the site of injury.

Five months postoperatively, the patient reported no pain (0/10) and possessed 95% of his normal function at this time. On physical examination, he was nontender to palpation along the joint line. There was no laxity with varus or valgus stress. He demonstrated 5/5 quadriceps strength with no evidence of an extensor lag. He had an active range of motion from 0 to 130 degrees of flexion, and there was no lag with straight leg raise. Repeat anterior-posterior and lateral X-rays demonstrated a well-reduced tibial tubercle fracture as well as well-positioned and nondisplaced hardware (Figure 6).

## 3. Discussion

Tibial tubercle avulsion fractures are a rare injury and can be associated with concomitant soft tissue damage, periosteal damage, and compartment syndrome leading to extensor mechanism disruption, joint laxity, or vascular compromise [6]. In a series of 336 tibial avulsion fractures in adolescent patients, there were 8 (2%) patellar or quadriceps tendon avulsions, 6 (2%) meniscal tears, 3 (1%) increased ligamentous laxity, and 12 (4%) compartment syndromes [8]. In this case report, we discuss a patient who suffered a tibial tubercle avulsion fracture with multiple serious concomitant injuries, a presentation which, to our knowledge, has not previously been reported in the literature.

Extensor mechanism deficit can occur concomitantly in patients with a tibial tubercle avulsion fracture due to patellar tendon rupture [9]. The reported incidence of patellar tendon rupture ranges from 2%–15.7% [8]. Limited range of motion due to pain or effusion from the avulsed tubercle can cause the variation in reported incidence. The presence of a palpable defect between the inferior pole of the patella and tibial tuberosity should heighten clinical suspicion for associated patellar tendon rupture [7]. If physical examination cannot be performed due to cast immobilization or pain, the presence of patella alta on radiographic imaging as well as calcified fragments below the patella may indicate the presence of patellar tendon rupture [7, 9]. The tibial tubercle represents the most inferior aspect of the extensor mechanism, and adolescents are at increased risk of injury due to the relative weakness of the physis compared to the tendon insertion [7]. The tibial tubercle ossifies in a systematic mechanism from the superior aspect of the epiphysis to the inferior margin [3]. The primary goal in treating this injury is to restore the extensor mechanism and, if the joint space is involved (as seen in types III and IV), to restore the integrity of the joint surface [10].

Isolated, noncomminuted tibial tubercle fractures (types IA, IB, and IIA) can be treated with closed reduction for 4–6 weeks, whereas tibial tubercle fractures that are comminuted or extend intraarticularly should be repaired via open reduction internal fixation [11]. Patients with a fracture pattern of the latter form should be evaluated for concomitant patellar ligament disruption, meniscal injury, or compartment syndrome [11]. In this case, suture anchors were placed along the anatomic insertion of the patellar tendon on the anterior tibial cortex. However, Howarth et al. describe a technique of patellar tendon reconstruction in an adolescent patient with a tibial tubercle fracture in which bioabsorbable suture anchors were placed above the growth plate on either side of the fracture site [12]. While this technique provides additional fixation on the proximal aspect of the fracture and assists with meniscal repair, it carries the risk of growth plate disruption or articular surface penetration. Therefore, this technique may need to be performed under fluoroscopic guidance in order to minimize these risks.

Compartment syndrome is a potentially devastating injury that can occur with tibial tubercle avulsion fracture due to soft tissue injury or the fractured component damaging the anterior tibial recurrent artery [4, 13]. Compartment syndrome is associated with fractures that extend proximally (Type II), fractures that encompass the entire proximal tibial physis (type IV), and displaced fractures [6, 11, 13]. In this case, our patient presented with a displaced fracture of the tibial tubercle that extended proximally into the joint space (type IIIB). Additionally, upon presentation to the ER and initial clinic visit, the patient had a stable neurovascular examination, which alleviated concern regarding compartment syndrome. Upon intraoperative examination of the injury site, it was noted that vascular damage was present which would have led to postoperative compartment syndrome if not addressed. While there lacks evidence to support an association between compartment syndrome and the type of tibial tubercle avulsion fracture and due to the variability in the temporal presentation of compartment syndrome, it is paramount that careful evaluation of vascular integrity and a low threshold for fasciotomy be in place to prevent vascular compromise.

94% of patients (248 total) return to their preinjury level at a mean of 28.9 weeks, 98% of patients (250 total) regained full knee range of motion at 22.3 weeks, and 99% of cases (334 out of 336) reported fracture union [8]. In this case, our patient reported 95% of normal function, near-full return of range of motion, and fracture union 20 weeks following operative management. Overall, there is a 28% reported complication rate following tibial tubercle avulsion fracture repair, with the most commonly reported complications being bursitis (56%) and tenderness overlying the tibial tubercle (18%) [8, 13]. In this instance, the patient did not suffer postoperative complications.

Previous reports of tibial tubercle avulsion fractures noted patients who had concomitant tendon avulsion, meniscal damage, ligament injury, and vascular compromise. However, there were no such reports of patients suffering multiple injuries in addition to a tubercle avulsion fracture. In this report, a patient who suffered a tibial tubercle avulsion fracture while jumping and colliding with another player had concomitant patellar tendon avulsion and subacute compartment syndrome that necessitated intraoperative fascial release. Due to the severity of compartment syndrome, each patient who undergoes ORIF should be evaluated at that time for compartment syndrome. In cases of tibial tubercle avulsion fracture, clinicians should have a high index of suspicion to evaluate for additional injuries that may be present.

This case report is limited in the duration of follow-up. As a 14-year old patient at the time of initial consultation, this patient was not skeletally mature at the time of follow-up. Tibial tubercle avulsion fractures can cause disruption to the growth plate which can cause skeletal deformities such as genu recurvatum or limb-length discrepancy, which can present in 4% and 5% of cases, respectively [8]. It is imperative to continue to follow up these patients until they have reached skeletal maturity to ensure normal growth without any resultant osseous deformities as additional procedures, such as growth plate modulation, may be required.

## 4. Conclusion

We present a unique case of a 14-year-old male who suffered a tibial tubercle avulsion fracture while jumping and colliding with another player during a basketball game. In addition to the tubercle fracture, this patient suffered patellar tendon avulsion and subacute compartment syndrome. Operative management included ORIF of the tibial tubercle and patellar tendon repair, as well as fascial release which was performed for impending compartment syndrome under arthroscopic guidance.
